# Case Report: Atypical extensive orbitofacial tuberculosis extending to the skull base and cavernous sinus revealed major histocompatibility complex class II deficiency in an 11-year-old girl

**DOI:** 10.3389/fped.2025.1663784

**Published:** 2025-11-04

**Authors:** Sameh Mezri, Ameni Amri, Sonia Essghaier, Karima Tlili, Maroua Rabhi, Mahdi Marmouri, Hager Barakizou

**Affiliations:** ^1^ ENT Department, Military Hospital of Tunis, Tunis, Tunisia; ^2^ Radiology Department, Military Hospital of Tunis, Tunis, Tunisia; ^3^ Pathology Department, Military hospital of Tunis, Tunis, Tunisia; ^4^ Pediatry Department, Military Hospital of Tunis, Tunis, Tunisia

**Keywords:** cavernous sinus, MHC class II deficiency, pediatric case report, orbital tuberculosis, paranasal sinus tuberculosis

## Abstract

**Background:**

Cavernous sinus tuberculosis is an extremely rare manifestation of central nervous system tuberculosis in children, with only two cases reported worldwide. It can mimic malignancy or other inflammatory conditions. Its occurrence in children with primary immunodeficiency, particularly major histocompatibility complex (MHC) class II deficiency, has not yet been described.

**Case report:**

We report an 11-year-old girl with a history of recurrent infections and chronic otitis media. She presented with right orbital swelling, severe headaches, and exophthalmos. Imaging revealed an extensive mass in the sinonasal and orbital regions, extending to the skull base and cavernous sinus. A computed tomography-guided biopsy and histopathology, supported by PCR testing for *Mycobacterium tuberculosis*, confirmed extensive orbital and cervicofacial tuberculosis. An immunological evaluation and genetic analysis revealed familial MHC class II deficiency. The patient received anti-tuberculosis therapy [isoniazid, rifampin, pyrazinamide, and ethambutol (HRZE) followed by isoniazid and rifampin (HR)], leading to clinical and radiological improvement. She continues with intravenous immunoglobulin replacement therapy every 21 days while awaiting a bone marrow transplantation.

**Conclusions:**

This case highlights the importance of considering tuberculosis in atypical cavernous sinus lesions in children, especially in endemic regions. Severe or unusual infections should prompt evaluation for underlying immunodeficiency.

## Introduction

Tuberculosis remains a major public health challenge worldwide, particularly in low- and middle-income countries. In these regions, both the pulmonary and extrapulmonary forms continue to affect vulnerable pediatric populations. While most pediatric tuberculosis cases affect the lungs, extrapulmonary involvement is rare and presents significant diagnostic and therapeutic challenges. Among these rare forms, cervicofacial tuberculosis involving the cavernous sinus and orbit is exceedingly uncommon and may mimic malignancy (1).

In immunocompetent children, *Mycobacterium tuberculosis* is usually contained within granulomas, which limit its spread. In contrast, children with compromised immunity may present with atypical disease forms, including disseminated or fulminant tuberculosis. Primary immunodeficiencies impair T-cell function and major histocompatibility complex (MHC) class II expression, increasing the risk of severe and recurrent infections caused by intracellular pathogens such as mycobacteria (2).

We report a unique case of extensive, atypical tuberculosis in an 11-year-old girl with familial MHC class II deficiency. Her initial presentation involved isolated orbital symptoms. The diagnosis was confirmed using histopathological and molecular investigations. This case emphasizes the importance of considering both tuberculosis and underlying primary immunodeficiencies in the differential diagnosis of atypical orbital masses in children. We aim to illustrate the diagnostic approach, radiological findings, therapeutic management, and the role of immunological evaluation in managing such complex cases.

## Case report

We present the case of an 11-year-old girl who had viral meningoencephalitis at age 5, resulting in unilateral sensorineural hearing loss. Since that episode, she had been followed for bilateral chronic otitis media with recurrent upper respiratory tract infections that were treated as needed with antibiotics, antipyretics, nasal lavage, and supportive care. Her immunizations were complete and age-appropriate, including the Bacillus Calmette–Guérin (BCG) vaccine.

The current episode began approximately 1 month prior to hospital admission, initially presenting as isolated right eyelid edema that was overlooked. Subsequently, she developed severe, persistent bifrontal and occipital headaches, prompting urgent evaluation.

On clinical examination, she had a right eyelid edema and grade III axial right-sided exophthalmos without limitation of ocular movements. Nasal endoscopy revealed inflamed mucosa with purulent discharge from the right superior meatus, consistent with active sinonasal inflammation.

The treating pediatrician prescribed oral amoxicillin-clavulanate (2 g/day for 8 days) and corticosteroids, nasal decongestants, nasal lavage, and oral mucolytics, suspecting acute ethmoiditis. As the symptoms did not show signs of improvement, a referral to our specialized hospital service was deemed necessary for further evaluation.

On admission, left jugulocarotid lymphadenopathy was noted, while the neurological examination was unremarkable. Her height was 137 cm (height-for-age z-score: −1.0) and her weight was 28 kg (weight-for-age z-score: −2.0), indicating she was underweight. Her BMI was 14.7 kg/m^2^ (BMI-for-age z-score: −2.2), consistent with moderate thinness. Historical records showed persistently low weight, supporting a diagnosis of chronic undernutrition.

**Figure 1:**
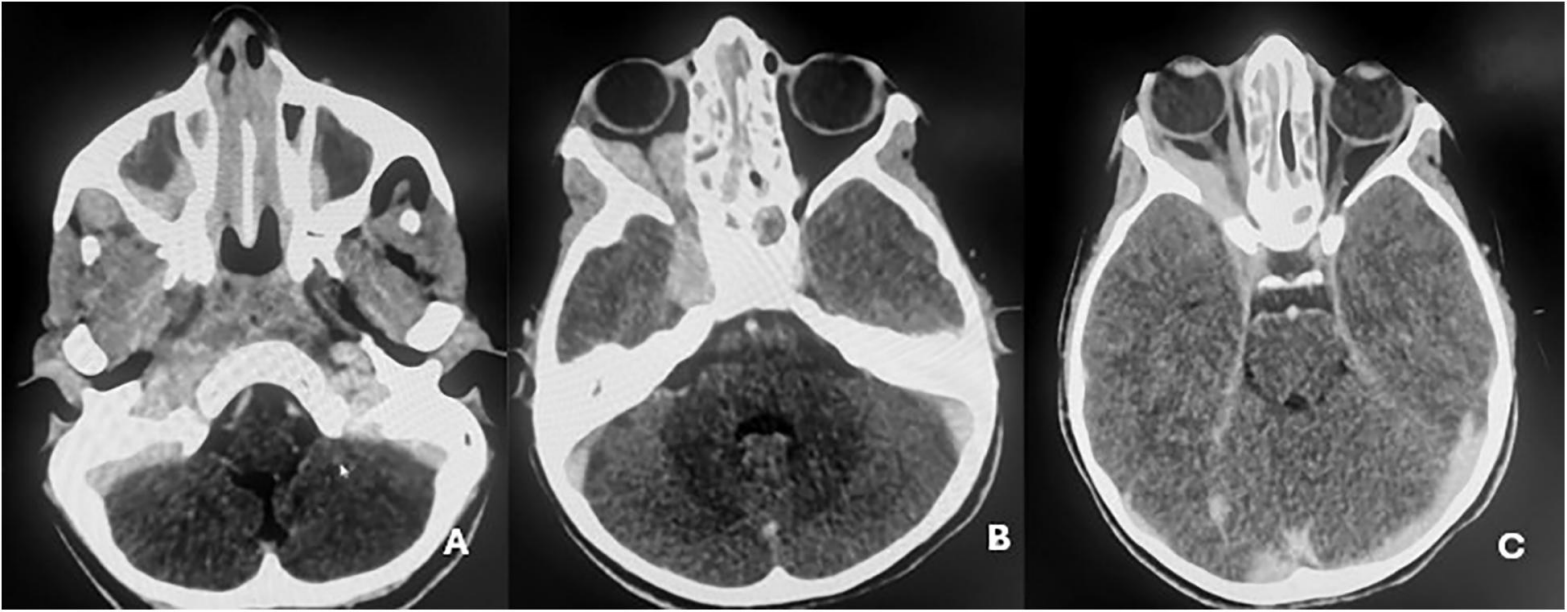
Contrast-enhanced cerebro-orbital scans demonstrating tissue expansion affecting both nasal cavities, extending into the maxillary sinuses **(A)**, and the right extraconal orbital space, extending to the ipsilateral cavernous sinus **(B,C)**.

**Figure 2:**
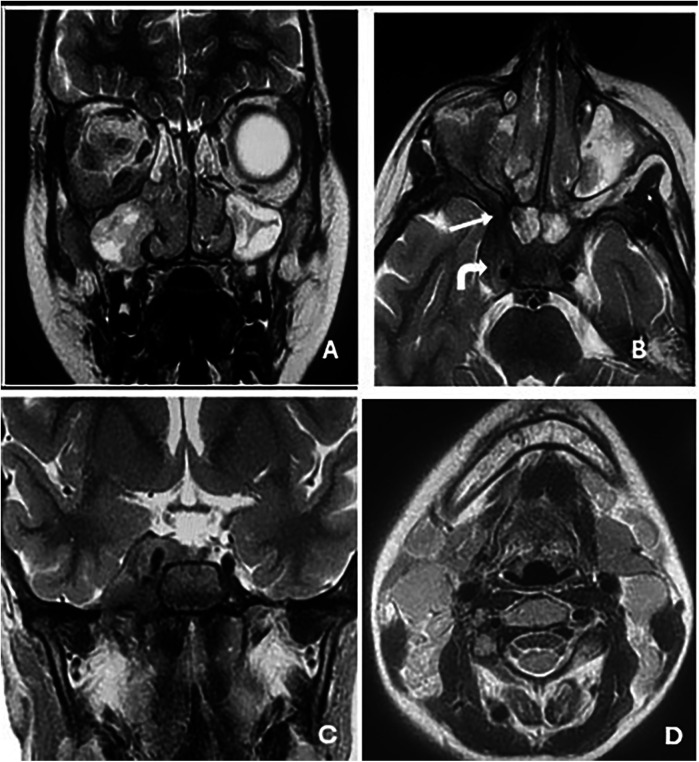
Orbital and cerebro-cervical MRI in T2-weighted sequences in the coronal **(A** and **C)** and axial **(B)** planes showing an expansive tissue signal process invading the right orbital extraconal space and nasal cavities **(A)**; the right cavernous sinus (solid arrow in **B**); and the pterygopalatine fossa, infratemporal fossa, and the foramina of the skull base, with **(B)** showing the right round and **(C)** the right oval. Scan showing non-necrotic bilateral jugulocarotid and spinal lymphadenopathy (**D**).

Contrast-enhanced CT of the brain and orbits demonstrated a tissue process involving both nasal cavities and maxillary sinuses, extending into the right extraconal orbital space and ipsilateral cavernous sinus (Figure 1). Orbital and cerebro-cervical MRI demonstrated an expansive tissue signal process affecting the right orbital extraconal space, nasal cavities, right cavernous sinus, pterygopalatine fossa, infratemporal fossa, and foramina of the skull base. Cervical lymphadenopathy was also present (Figure 2).

**Figure 3:**
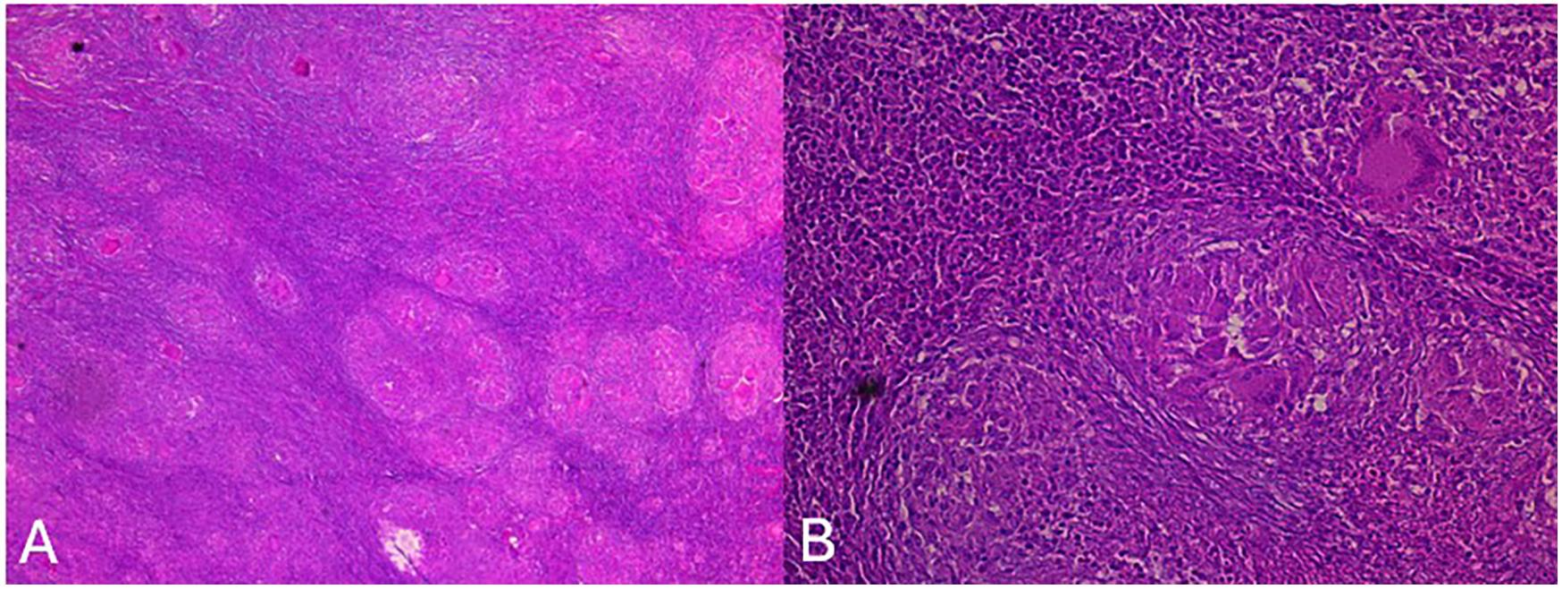
Anatomopathological study showing (HEx4) lymph node parenchyma altered by a specific inflammatory reaction with multiple tuberculous granulomas **(A)** and (HEx20) an epithelioid granuloma with focal central caseous necrosis and Langhans-type giant cells **(B)**.

To exclude lymphoma, a thoraco-abdomino-pelvic CT scan was performed and revealed no distant lesions. A CT-guided biopsy of the infratemporal mass, along with lymph node excision, was undertaken. The histopathological examination demonstrated an epithelioid granulomatous reaction with multinucleated giant cells and central caseating necrosis, highly suggestive of tuberculosis (Figure 3). PCR testing confirmed the presence of *M. tuberculosis,* whereas the sputum analysis for acid-fast bacilli (AFB) was negative.

Pre-treatment evaluations included an assessment of acetylator status, complete blood count, renal and liver function, and serum electrolytes. The patient was initiated on standard anti-tuberculosis chemotherapy.

In view of the patient's atypically extensive tuberculosis presentation and a history of recurrent pulmonary and gastrointestinal infections in her older sister, an immunodeficiency workup was undertaken.

The quantitative analysis demonstrated profound CD4+ T-cell lymphopenia with IgA and IgG hypogammaglobulinemia, while her IgM levels remained within the normal range. Flow cytometry revealed a markedly reduced CD4/CD8 ratio (<0.3).

Peripheral blood mononuclear cells were isolated via Ficoll density gradient, stimulated with phytohemagglutinin (PHA), and labeled with monoclonal antibodies at the specialized immunology laboratory of the Pasteur Institute of Tunis. The analysis demonstrated the near-complete absence of human leukocyte antigen – DR isotype (HLA-DR) expression on activated lymphoblasts (2% positive), whereas the activation marker CD25 was expressed in 99% of the cells, indicating adequate lymphocyte activation. These findings confirmed MHC class II deficiency, consistent with a combined immunodeficiency due to defective HLA class II expression (Table 1).

**Table 1:** Immunological profile of the patient and the flow cytometry analysis results.

Parameter	Result	Reference range
IgG (g/L)	4	7–13
IgA (g/L)	<0.2	0.7–2.8
IgM (g/L)	1.7	0.4–2.3
CD4+ T cells (cells/µL)	130	500–1,200
HLA-DR on PHA-stimulated lymphoblasts	2% positive	>90%
CD25 on PHA-stimulated lymphoblasts	99% positive	—

Similar immunological findings were observed in the patient's older sister, confirming familial primary immunodeficiency due to MHC class II deficiency (bare lymphocyte syndrome type II).

**Figure 4:**
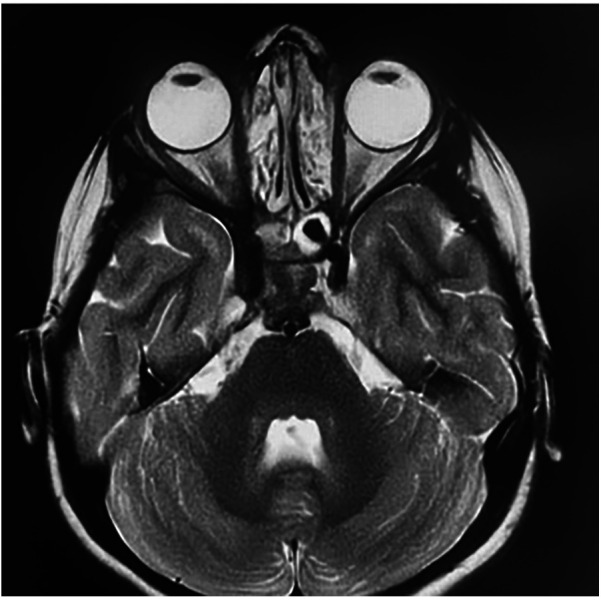
MRI (after 6 months of treatment) showing regression of the previously described lesions.

The patient is currently receiving intravenous immunoglobulin replacement therapy every 21 days while awaiting allogeneic hematopoietic stem cell transplantation (HSCT). After 6 months of anti-tuberculosis therapy, comprising 3 months of quadruple therapy (isoniazid, rifampicin, pyrazinamide, and ethambutol) followed by 3 months of dual therapy (isoniazid and rifampicin), clinical follow-up demonstrated complete resolution of her exophthalmos. Subsequent MRI confirmed marked regression of the lesions (Figure 4). At the time of reporting, she was in her ninth month of treatment, exhibiting no evidence of drug resistance and showing progressive weight gain.

## Discussion

Cavernous sinus tuberculosis (CST) remains an exceptionally rare condition in children, with only two pediatric cases reported to date. Familial MHC class II deficiency, also referred to as bare lymphocyte syndrome type II, is a rare autosomal recessive primary immunodeficiency disorder, with roughly 150 cases documented worldwide (3). The simultaneous occurrence of these two uncommon conditions in an 11-year-old patient represents an extraordinarily rare clinical scenario.

Tuberculosis affecting the central nervous system (CNS) represents one of the most severe forms of extrapulmonary tuberculosis in children, with a clinical spectrum ranging from tuberculous meningitis (TBM) to compressive myelopathy secondary to vertebral involvement, as well as the development of tuberculomas (4).

The cavernous sinus is an irregularly shaped, endothelium-lined venous structure positioned on either side of the sella turcica. It is located laterally and superiorly to the sphenoid sinus and immediately posterior to the optic chiasm (5). Venous drainage occurs from the central face and frontal sinuses. A notable anatomical characteristic is the absence of valves in its connecting veins, permitting bidirectional blood flow and thereby increasing the risk of infectious spread and thrombosis (5).

To date, only two pediatric cases of cavernous sinus tuberculoma have been documented. The first, reported in 2007, involved a 3-year-old Thai patient who presented with progressive cavernous syndrome, including right eyelid ptosis, mild proptosis, medial gaze palsy, and eventual total ophthalmoplegia. The initial clinical diagnosis was acute sphenoid sinusitis. The tuberculoma diagnosis was inferred from a reactive tuberculin test, an abnormal chest radiograph showing bilateral reticular infiltration and a calcified nodule, and a positive *M. tuberculosis* culture from gastric contents. No direct biopsy of the cavernous sinus was performed. The patient achieved complete recovery following anti-tuberculosis therapy (6) (Table 2).

**Table 2:** Reported pediatric cavernous sinus tuberculoma cases: clinical features and outcomes.

Case number/ age (source)	Clinical presentation	Diagnostic methods	Key findings	Treatment	Outcome
1: 3 years (6)	Progressive cavernous syndrome (drooping right eyelid, mild proptosis, medial gaze palsy, and total ophthalmoplegia), with initially acute sphenoid sinusitis	Abnormal chest film, reactive tuberculin test, and positive *M. tuberculosis* culture from gastric contents. Neuroimaging (CT, MRI) showed non-specific mucoperiosteal thickening of the sphenoid sinuses and left cavernous sinus enhancement. Biopsy not allowed	Tuberculoma inferred from the therapeutic response. Granulomatous mass in the left upper lung	Anti-tuberculosis agents (specific regimen not detailed)	Complete recovery
2: 11 years (7)	Right hemicranial headache, diplopia, right facial numbness (V1–V2), and chronic ear discharge	MRI, CT, CSF analysis, and surgical biopsy with histopathology	Lesion in the right cavernous sinus and Meckel's cave, petrous apex erosion, *Acinetobacter* in ear pus, and tuberculous granuloma on histopathology	IV antibiotics (cephalosporin + aminoglycoside), surgical resection, and anti-tuberculosis therapy	Resolution of symptoms, complete radiological response at 12 months, and continued treatment to 18 months

The second case, reported in 2014, involved an 11-year-old patient with a 2-year history of untreated right ear discharge and hearing loss. She presented with right hemicranial headache, diplopia, and facial numbness. The clinical examination revealed right VI cranial nerve palsy and V1–V2 hypoesthesia, accompanied by purulent otorrhea. MRI identified a lesion in the right cavernous sinus and Meckel's cave with erosion of the petrous apex. The lesion was initially suspected to be an inflammatory granuloma secondary to chronic otitis media. A definitive diagnosis was established postoperatively when histopathology demonstrated caseating granulomas consistent with tuberculosis. The patient received anti-tuberculosis therapy following a near-total surgical resection. MRI performed after 12 months confirmed complete resolution of the lesion, and the therapy was continued for a total of 18 months (7) (Table 2).

A cavernous sinus tuberculoma poses a considerable diagnostic challenge, as it may mimic more common conditions, including meningioma, lymphoma, sarcoidosis, or bacterial and fungal infections (3). This similarity frequently leads to diagnostic delays, with empirical therapies sometimes initiated before the commencement of anti-tuberculosis treatment, potentially worsening patient outcomes—particularly in anatomically critical sites such as the CNS (4).

The diagnostic evaluation of CST relies on a combination of imaging and microbiological confirmation. Contrast-enhanced CT and MRI are essential for identifying lesions within the cavernous sinus (3), and MRI can detect early infarcts, basal ganglia enhancement, and basal cistern exudates (8). Nevertheless, imaging findings are often non-specific and may resemble other intracranial masses (3). Direct microbiological confirmation from the affected site remains the gold standard. Due to the paucibacillary nature of pediatric tuberculosis and the challenges in obtaining adequate CNS samples, a definitive confirmation can be difficult (4). The recovery of *M. tuberculosis* from cerebrospinal fluid (CSF) is typically low, and culture growth is slow (4). Molecular assays, such as the Xpert MTB/RIF test, are recommended for rapid detection and drug-resistance evaluation, although their sensitivity can vary (8). When feasible, tissue biopsy provides definitive confirmation, demonstrating caseating granulomas and allowing subsequent culture and molecular analyses (3). In adult patients, some CST cases were only conclusively diagnosed after surgical resection (9).

Children affected by paucibacillary tuberculosis, which is characterized by a low bacterial load, can nevertheless mount a robust immunological response, resulting in granuloma formation. This contrast creates a diagnostic paradox: although the host immune system attempts to contain the infection through granuloma formation, the low bacterial burden complicates direct detection of *M. tuberculosis* (8).

In individuals with MHC class II deficiency, the immunological hallmark is the absent or markedly reduced expression of MHC class II molecules (HLA-DR, HLA-DQ, and HLA-DP) on antigen-presenting cells (APCs), including B cells, monocytes, and dendritic cells (3). This defect impairs both cellular and humoral immunity, as these molecules are critical for the development, maturation, and functional activation of CD4+ T helper (Th) cells (3). Patients typically present with severe CD4+ T-lymphocytopenia, an inverted CD4:CD8 ratio, and hypogammaglobulinemia, reflecting impaired Th cell-dependent antibody production by B cells (3). Altered thymic selection of CD4+ T cells further exacerbates this immune defect (10).

CD4+ T cells, activated via MHC class II molecules on APCs, are central to the host's resistance against *M. tuberculosis*. They orchestrate granuloma formation and maintenance, which contain mycobacteria. In MHC class II deficiency, impaired antigen presentation leads to weak CD4+ T-cell activation and function, explaining the persistent infection and atypical disease dissemination (11, 12). Consequently, granulomas may be present but functionally inadequate, emphasizing the importance of histopathology not only for diagnosis but also for evaluating granulomatous responses in immunodeficient patients.

Typically, an 11-year-old child would have a lower risk of progression to active tuberculosis and would present with mild clinical manifestations. In this patient, the severe CST presentation reflects her underlying primary immunodeficiency. The “Th1 skew” and lymphocyte predominance generally observed in immunocompetent children are markedly compromised in MHC class II deficiency (13).

The management of patients with such conditions requires prolonged and carefully monitored therapy. In young children, higher drug dosages may be necessary to achieve effective bactericidal activity (14). In this patient, the presence of atypical CNS involvement, including the skull base and cavernous sinus, combined with MHC class II deficiency, warranted a 12-month anti-tuberculosis regimen, in line with international guidelines for CNS and osteoarticular tuberculosis (15).

Corticosteroids are frequently administered in children with CNS tuberculosis, particularly those with tuberculous meningitis, to reduce inflammation, cerebral edema, and intracranial pressure (15, 16). In patients living with primary immunodeficiency, corticosteroid use presents a therapeutic challenge, as these agents may suppress immune function while simultaneously mitigating severe inflammatory complications or immune reconstitution inflammatory syndrome (IRIS) (17, 18).

Additional clinical considerations include drug interactions, particularly with rifamycins, which induce cytochrome P450 enzymes and drug transporters, potentially reducing serum concentrations of co-administered immunosuppressants or other therapies required to manage complications associated with MHC class II deficiency (19, 20).

HSCT remains the only curative therapy for individuals with MHC class II deficiency (3). However, active CNS tuberculosis can complicate transplant eligibility. Achieving effective infection control prior to HSCT is essential for optimizing transplant outcomes and minimizing post-transplant complications (10).

## Conclusion

This case highlights cavernous sinus tuberculosis in a child with MHC class II deficiency. Tuberculosis should be considered in atypical intracranial lesions, and severe or unusual infections should prompt evaluation for underlying immunodeficiency.

## Data Availability

The original contributions presented in the study are included in the article/Supplementary Material, further inquiries can be directed to the corresponding author.
